# Book Reviews

**Published:** 1874-01-15

**Authors:** 


### Book Reviews.

TREATISE on the Diseases of the Eye. By J. Saelberg Wells, F.R.C.S., Professor of Ophthalmology in King's College, London, Surgeon to the Royal London Ophthalmic Hospital, Moorsfield, etc., etc. Second American, from the third English, edition. Philadelphia: Henry C. Lea, 1873.

No better evidence of the value of this work is needed than the fact that, since its first appearance, in 1868, it has had three English editions and two American, and has been translated into the German and French languages. Voluminous as has the literature of ophthalmology become since the ophthalmoscope was devised, this work merits the designation of being the most valuable one in the English language for the general practitioner. The diseases are treated of in a clear, concise manner; and the extensive field of observation afforded the author, as a member of the surgical staff of the Royal London Ophthalmic Hospital, has given ample opportunity to test the correctness of his views.

His suggestions regarding the variations of refraction of the eye, and the proper mode of correcting them, are explicit, and well calculated to correct many of the popular errors regarding the use of spectacles. For his caution against the unscientific effort of opticians and jewelers to adjust glasses to correct these variations, as dangerous, he merits the thanks of the public, who are often astonished when informed of the risks they thus incur.

The addition, to the American edition, of selections from the test-types of Jaeger and Snellen, will be found a convenience to many.

The six chromo-lithographic plates, representing pathological conditions of the interior of the eye, are well executed, and faithfully represent those structural changes. In short, the work fills a place of usefulness.

